# Supplementary material for: Genome-Wide Analysis of the SRPP/REF Gene Family in Taraxacum kok-saghyz Provides Insights into Its Expression Patterns in Response to Ethylene and Methyl Jasmonate Treatments
Source: Int J Mol Sci. 2024 Jun 22;25(13):6864. doi: 10.3390/ijms25136864 (PMC11241686; doi:10.3390/ijms25136864)
Supplement: Supplementary file 1 [file ijms-25-06864-s001.zip › Figure S1 TkSRPPREF gene family protein tertiary structure.pdf]

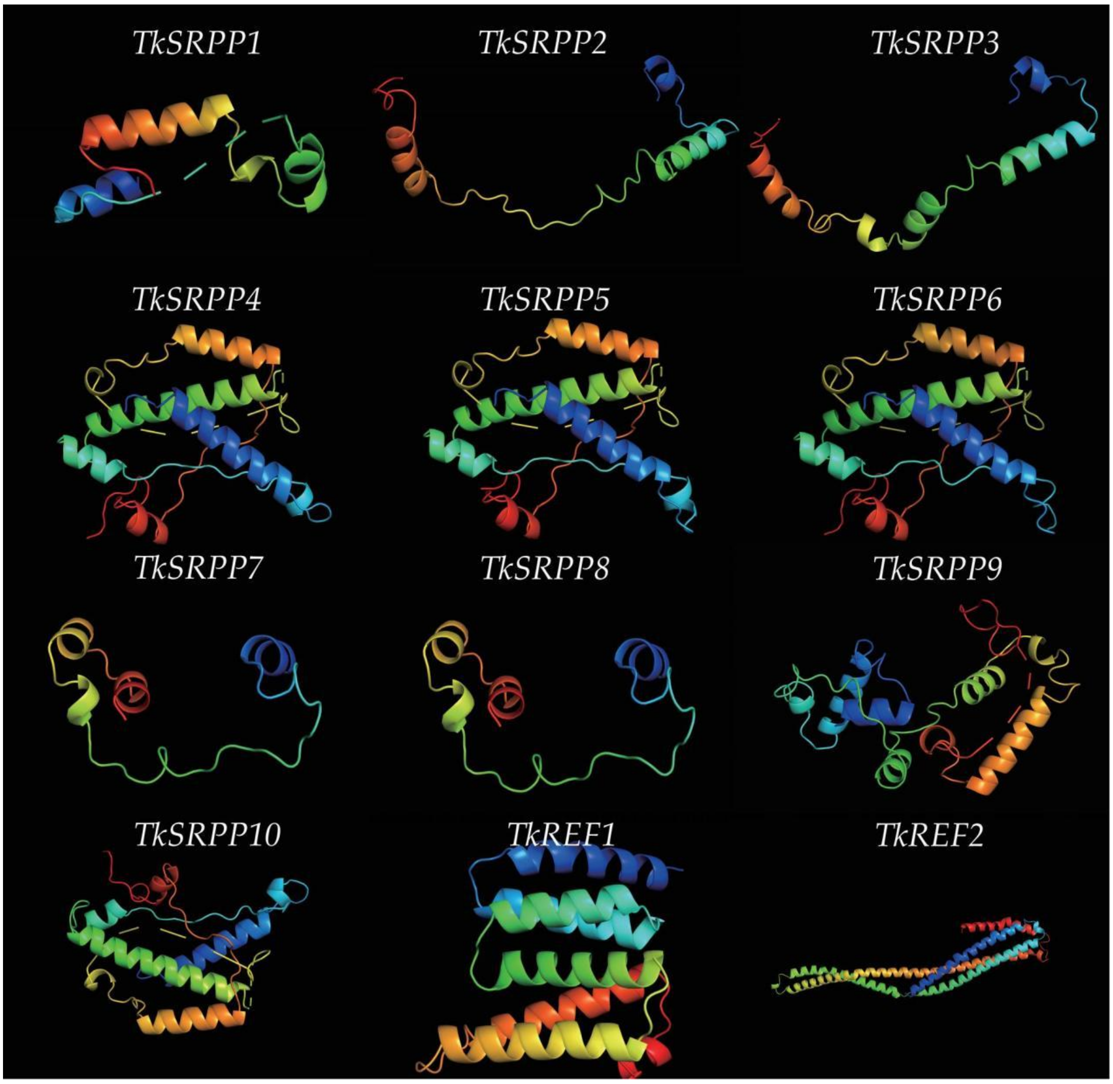

Figure S1. The 3D structure modeling of *TkSRPP/REF* proteins.The 3D structures were colored by rainbow order, representing N to C terminus.
