# Supplementary material for: Genome-Wide Analysis of the SRPP/REF Gene Family in Taraxacum kok-saghyz Provides Insights into Its Expression Patterns in Response to Ethylene and Methyl Jasmonate Treatments
Source: Int J Mol Sci. 2024 Jun 22;25(13):6864. doi: 10.3390/ijms25136864 (PMC11241686; doi:10.3390/ijms25136864)
Supplement: Supplementary file 1 [file ijms-25-06864-s001.zip › Figure S2 Phylogenetic analysis of TkSRPPREF in multiple species.pdf]

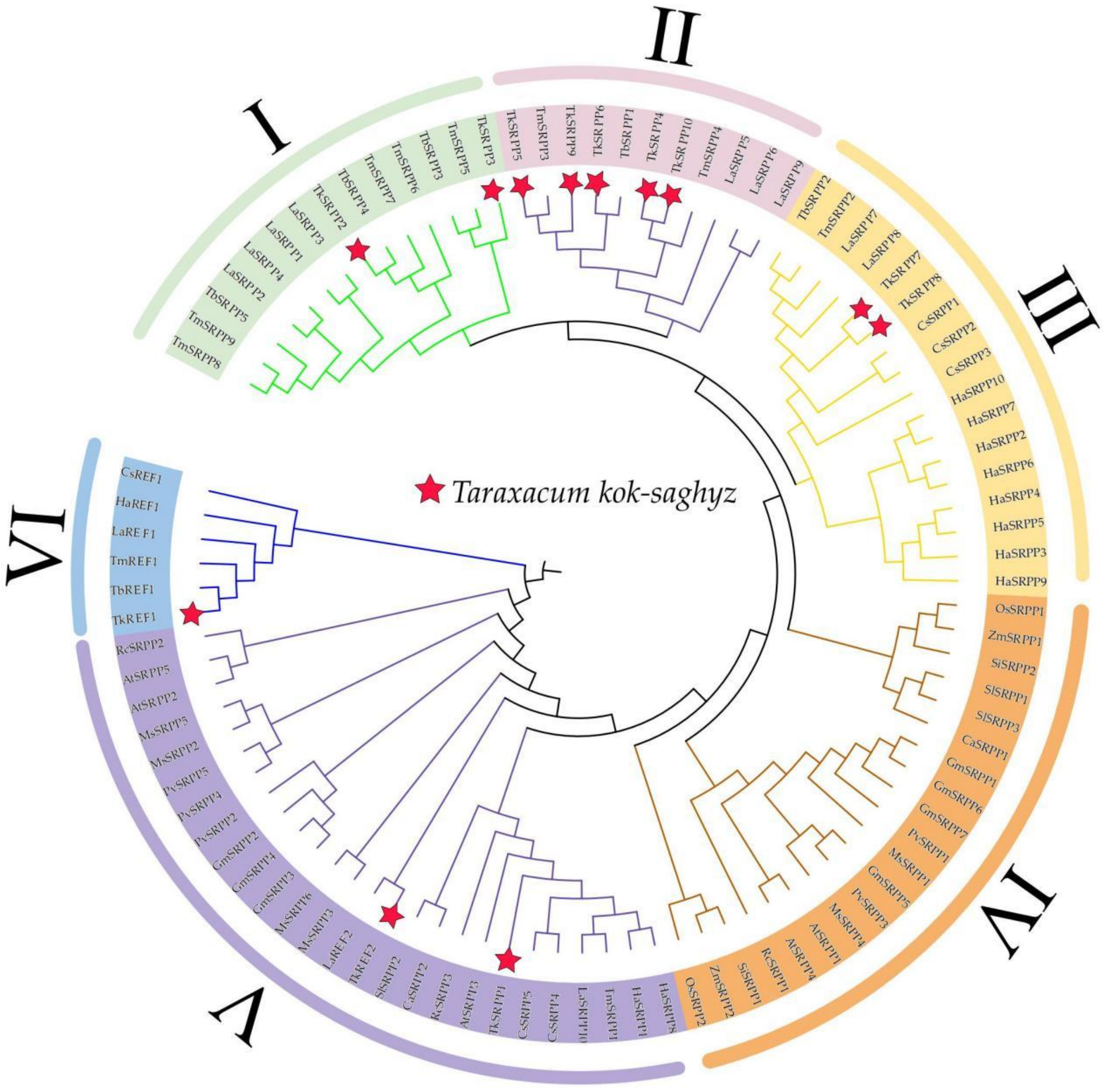

Figure S2. Phylogenetic analysis of *TkSRPP/REF* in multiple species.The putative SRPP/REF genes from *T. mongolicum* , *L. sativa* , *H. annuus* , *G. max* , *T. brevicorniculatum* , *C. annuum* , *S. lycopersicum* , *C. scolymus* , *O. sativa* , *Z. mays* , *A. thaliana* , *P. vulgaris* , *M. truncatula* and *R. communis* were identified to construct phylogenetic tree.Phylogenetic trees were plotted using the neighbor-joining (NJ)method with a bootstrap value of 1000. Eighty-two genes were divided into five clades (Ⅰ–Ⅵ) and identified with different colors. The red pentagram represents *T. kok-saghyz*.
