# Supplementary material for: Genome-Wide Analysis of the SRPP/REF Gene Family in Taraxacum kok-saghyz Provides Insights into Its Expression Patterns in Response to Ethylene and Methyl Jasmonate Treatments
Source: Int J Mol Sci. 2024 Jun 22;25(13):6864. doi: 10.3390/ijms25136864 (PMC11241686; doi:10.3390/ijms25136864)
Supplement: Supplementary file 1 [file ijms-25-06864-s001.zip › Figure S3 Gene structure ,conserved domain and protein motif of the SRPPREF gene family in Taraxacum kok-saghyz.pdf]

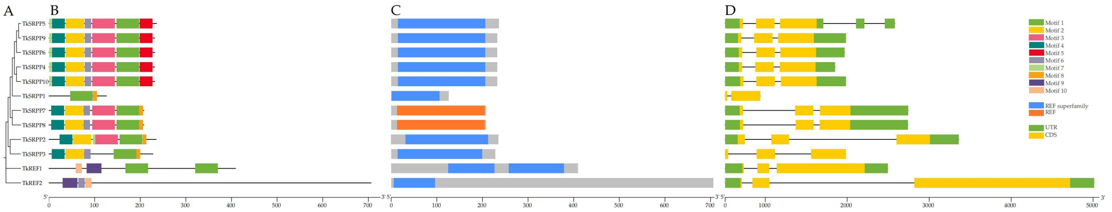

Figure S3. Gene structure ,conserved domain and protein motif of the *SRPP/REF* gene family in *Taraxacum kok-saghyz*.(A)The phylogenetic tree was constructed based on the full-length sequences of *TkSRPP/REF* proteins.(B)Conserved motifs of *TkSRPP/REF* proteins. Motifs with specific colors can be found on the respective *TkSRPP/REF* proteins. The order of the motifs corresponds to their position within individual protein sequences.(C)Conserved Tk*SRPP/REF* domains.(D)The intron-exon organizations of T*kSRPP/REF* genes. Yellow boxes indicate UTR; green boxes indicate CDS;black lines indicate introns.
