# Supplementary material for: Genome-Wide Analysis of the SRPP/REF Gene Family in Taraxacum kok-saghyz Provides Insights into Its Expression Patterns in Response to Ethylene and Methyl Jasmonate Treatments
Source: Int J Mol Sci. 2024 Jun 22;25(13):6864. doi: 10.3390/ijms25136864 (PMC11241686; doi:10.3390/ijms25136864)
Supplement: Supplementary file 1 [file ijms-25-06864-s001.zip › Figure S4 Expression profile of TkSRPPREF gene in Taraxacum kok-saghy leaves under ethylene induced hormone treatment.pdf]

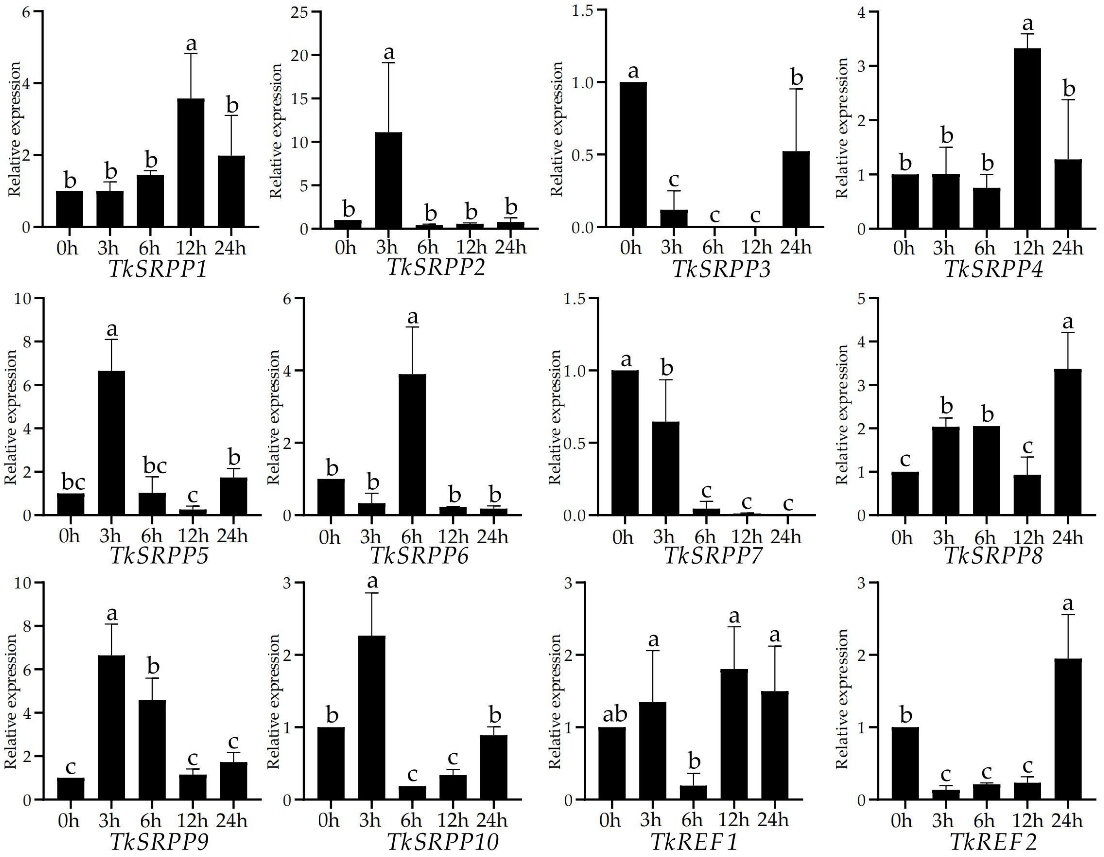

Figure S4. Expression profile of *TkSRPP/REF* gene in *Taraxacum kok-saghy* leaves under ethylene induced hormone treatment. The data is the average ± SD of three biological replicates. The error bar displays the mean ± SE of three independent replicates. The average values represented by the same letter showed no significant difference when p<0.05, as determined by Duncan's multiple range test.
