# Supplementary material for: Genome-Wide Analysis of the SRPP/REF Gene Family in Taraxacum kok-saghyz Provides Insights into Its Expression Patterns in Response to Ethylene and Methyl Jasmonate Treatments
Source: Int J Mol Sci. 2024 Jun 22;25(13):6864. doi: 10.3390/ijms25136864 (PMC11241686; doi:10.3390/ijms25136864)
Supplement: Supplementary file 1 [file ijms-25-06864-s001.zip › Table S1.Prediction of TkSRPPREFs protein secondary structure in Taraxacum kok-saghyz..pdf]

Table S1. Prediction of TkSRPP/REFs protein secondary structure in Taraxacum kok-saghyz.

Protein	Alpha helix (%)	Beta turn (%)	Random coil (%)	Distribution of secondary structure elements
TkSRPP1	56.35	8.73	25.40	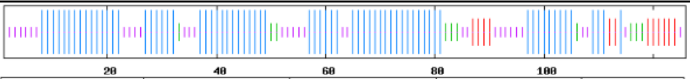
TkSRPP2	62.55	3.40	29.36	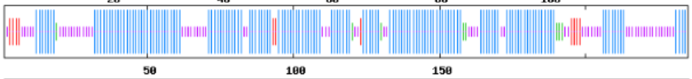
TkSRPP3	60.09	3.95	32.02	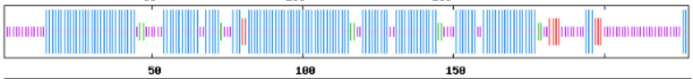
TkSRPP4	62.07	6.03	25.00	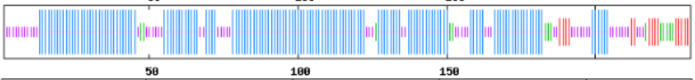
TkSRPP5	61.02	2.54	29.66	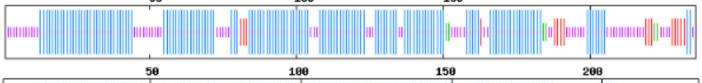
TkSRPP6	62.50	3.02	28.45	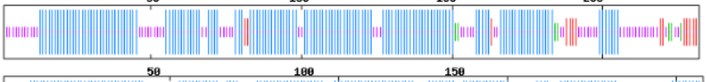
TkSRPP7	70.19	4.33	23.08	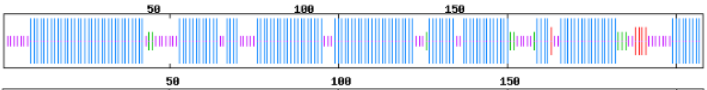
TkSRPP8	72.12	2.40	23.56	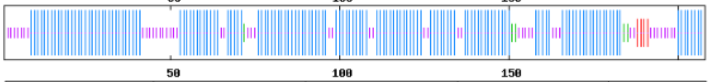
TkSRPP9	62.50	6.03	23.71	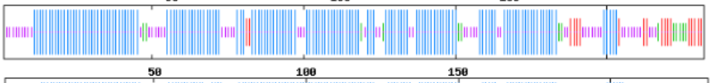
TkSRPP10	62.07	6.03	25.00	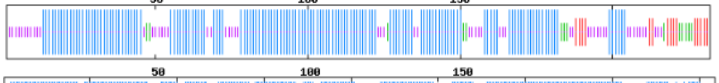
TkREF1	76.04	4.16	14.67	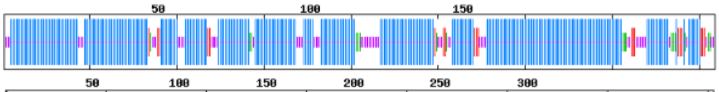
TkREF2	66.86	5.24	17.85	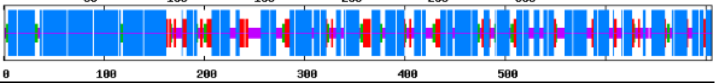
